# A Leaky Blood–Brain Barrier to Fibrinogen Contributes to Oxidative Damage in Alzheimer’s Disease

**DOI:** 10.3390/antiox11010102

**Published:** 2021-12-31

**Authors:** James G. McLarnon

**Affiliations:** Department of Anesthesiology, Pharmacology and Therapeutics, Faculty of Medicine, The University of British Columbia, 2176 Health Sciences Mall, Vancouver, BC V6T1Z3, Canada; mclarnon@mail.ubc.ca

**Keywords:** fibrinogen, fibrin aggregation and clots, blood–brain barrier, reactive oxygen species, neurodegeneration, amyloid-β peptide, microglia, neurovascular unit

## Abstract

The intactness of blood–brain barrier (BBB) is compromised in Alzheimer’s disease (AD). Importantly, evidence suggests that the perturbation and abnormalities appearing in BBB can manifest early in the progression of the disease. The disruption of BBB allows extravasation of the plasma protein, fibrinogen, to enter brain parenchyma, eliciting immune reactivity and response. The presence of amyloid-β (Aβ) peptide leads to the formation of abnormal aggregates of fibrin resistant to degradation. Furthermore, Aβ deposits act on the contact system of blood coagulation, altering levels of thrombin, fibrin clots and neuroinflammation. The neurovascular unit (NVU) comprises an ensemble of brain cells which interact with infiltrating fibrinogen. In particular, interaction of resident immune cell microglia with fibrinogen, fibrin and Aβ results in the production of reactive oxygen species (ROS), a neurotoxic effector in AD brain. Overall, fibrinogen infiltration through a leaky BBB in AD animal models and in human AD tissue is associated with manifold abnormalities including persistent fibrin aggregation and clots, microglial-mediated production of ROS and diminished viability of neurons and synaptic connectivity. An objective of this review is to better understand how processes associated with BBB leakiness to fibrinogen link vascular pathology with neuronal and synaptic damage in AD.

## 1. Introduction

Alzheimer’s disease (AD) is manifest as deficiencies in cognitive function and memory arising from abnormalities in neuronal and synaptic signaling in diseased brain [1]. In addition to neuronal and synaptic dysfunction, AD brain exhibits overt changes in other cellular functional processes including immune cell mobilization, activation and response. In particular, microglial activation in response to deposits of amyloid peptide (Aβ) characterize an environment of chronic inflammation in AD brain. Products of microglial activation, such as pro-inflammatory chemokines and cytokines and reactive oxygen species (ROS), contribute to the overall progressive neurodegeneration evident in AD brain [2].

Abnormalities in properties of cerebral vasculature represent another characteristic property inherent in AD brain. Individuals with AD commonly present a reduced cerebral blood flow (CBF) relative to non-demented (ND) controls [3]. Considerable structural perturbations of microvessels have also been reported in AD brain [4,5,6]. Animal models of AD have demonstrated marked abnormalities in the shapes and structures of microvessels with similar perturbations to those reported in human AD tissue [6]. Faulty angiogenesis has also been considered as a putative neurotoxic process in AD [7], with anti-angiogenic treatment found neuroprotective by stabilizing neurovascular remodeling in an animal model of AD [8]. At present, the links between vascular pathology, chronic inflammation and the loss of cognition and memory in AD brain remain elusive and largely unestablished.

The irregularities and abnormalities in vasculature are associated with an increased permeability of the blood–brain barrier (BBB) in AD. A compromised and leaky BBB is a characteristic feature of AD brain [1,9]. Indeed, evidence using different imaging modalities suggests that an increased permeability of BBB constitutes a relatively early event in the disease progression [10,11]. Presumably, the leakiness of BBB is correlated to some manner and extent with the perturbations of blood vessels noted above and the diminished CBF in AD brain. Importantly, the loss in integrity of BBB is associated with infiltration of plasma proteins into brain. The influx of plasma factors through a disrupted BBB could serve as a potent stimulus for immune cell response to protect against neurotoxicity arising from pathogen invasion. 

The intrusion of fibrinogen through a compromised BBB has received considerable attention with relevance to the pathogenesis of AD [12]. Early animal model studies reported elevated levels of fibrinogen and/or fibrin (termed fibrin(ogen) in brain parenchyma of transgenic mice [13,14] and in Aβ42-injected rat hippocampus [15]. Concomitant abnormalities in BBB function were also demonstrated with the entry of Evan’s blue dye in AD mice brain [13] and immunoglobulin (IgG) in peptide-injected rat brain [16]. A common finding from the different animal model studies was that infiltrating fibrin(ogen) showed evidence for co-localization with deposits of Aβ in brain parenchyma. 

The difficulties in the interpretation and translation of results from AD animal model studies to the human disease condition have recently been considered [17]. In this regard, to help validate animal model data on fibrinogen entry through a damaged BBB, postmortem brain tissue from AD patients and control ND (non-demented) individuals has been examined. Levels of fibrinogen were found to be markedly elevated in AD, relative to ND, brain samples from the entrohinal cortex [15]. Furthermore, double staining protocols showed evidence for diffuse fibrinogen immunoreactivity (ir) to be in close association with Aβ deposits in parenchymal regions. Overall, human AD brain was characterized by leakiness of BBB, infiltration of fibrinogen into parenchyma and localization of the plasma protein in regions containing Aβ peptide. Fibrin(ogen) extravasation has also been studied in frontal cortical and hippocampal regions of human postmortem brain tissue [18]. All regions of AD tissue showed marked increases in levels of fibrin(ogen) relative to ND samples. Overall, fibrin(ogen) infiltration through a porous BBB in human AD tissue closely replicated the findings from animal model work.

Subsequent studies have reported specificity in fibrinogen association and binding with Aβ [19]. Importantly, a consequence of the interaction of fibrinogen or fibrin with peptide was an effect to cause abnormal fibrin clots [20]. In essence, the results suggested structural changes in clots induced by Aβ caused an increased resistance to thrombotic enzymatic degradation and an enhanced persistence of fibrin in brain [20,21]. The presence of peptide deposits also added another complication in vascular modulation. It was found that Aβ interacted with the contact system and circulating coagulation factor XII to alter the coagulation process and the thrombin-dependent formation of fibrin clots [18,22]. This process could possibly cause an occlusion of blood capillaries. Activation of factor XII also leads to upregulation of bradykinin via another arm of the contact system [20]. Thus, Aβ modulation of the activation of the factor XII-dependent contact system in microvessels and in brain parenchyma with leaky BBB could contribute to neuroinflammation and neurodegeneration in AD.

The neurovascular unit (NVU) is an appropriate cellular ensemble to examine the overall effects of fibrinogen leakage into brain on cellular function and response. The NVU represents a network of interactive brain cells comprising neurons, astrocytes, microglia, oligodendrocytes and elements of vasculature including endothelial cells and pericytes The NVU offers a conceptual framework for characterizing interactions between its members and for studying mechanisms of actions of effectors such as fibrinogen and Aβ on properties of cells. Additionally, the NVU has utility in elucidating autocrine actions of cells. In this case, cells expressing receptors for their own secretory products can demonstrate an amplification of response within the NVU network.

### Objectives 

The overall goal of this review is to provide a framework linking fibrinogen passage through a disrupted BBB to neuronal and synaptic degeneration in AD. A specific objective is to document coupling of fibrinogen leakage to increased microglial-derived ROS which is neurotoxic in AD brain (see Section 3.6). The review presents a summary of findings of fibrinogen permeability through a leaky BBB in animal model studies. To help verify the interpretation of animal model data, work done on human AD brain tissue is reviewed. A following sub-section then considers the effects of fibrinogen interaction with Aβ which leads to the formation of abnormal fibrin clots. The effect of pharmacological modulation of fibrinogen binding to Aβ is then briefly discussed. Fibrinogen, fibrin and their association with Aβ also alter the contact factor coagulation process and this point is next reviewed. A following main section then considers the effects of fibrinogen infiltration into brain on individual members of the NVU. Selected relevant work briefly summarizes fibrinogen interactions with microglia, astrocytes, neurons, endothelial cells and pericytes. A final section then considers studies which have examined the coupling between fibrinogen passage into brain parenchyma through a damaged BBB and reactive oxygen species (ROS) neurotoxicity.

The term fibrin(ogen) is commonly used in the literature in cases where fibrinogen or fibrin (or both) could be the primary factor acting to cause neuroinflammation and neurodegeneration in AD. This convention is used in the review.

## 2. Selected Relevant Studies

### 2.1. Fibrinogen Infiltration into Brain

Several studies have initially documented fibrinogen extravasation through a compromised BBB in transgenic AD animal models [13,14] and in Aβ42-injected rat hippocampus [15]. Overall, considerable fibrinogen immunoreactivity (ir) was evident in brain parenchyma, suggesting an increased BBB permeability in AD mice and in rats injected with Aβ42 relative to controls. Furthermore, cerebral microvessels in transgenic mice and in Aβ42-injected rats exhibited abnormalities in shape and structure suggestive of a disrupted BBB. A noteworthy finding from AD animal model study was that fibrin(ogen) showed co-localization and association with deposits of Aβ in parenchymal regions of brain.

The defibrinogenating compound, ancrod, was employed to examine the effects of reducing circulating levels of fibrinogen in animal model studies. Ancrod treatment was found effective in attenuating vascular pathology in transgenic animals [13]. The authors postulated that reducing fibrinogen acted to dampen microglial responses to the plasma protein leading to improved BBB function and diminished levels of neuroinflammation. In Aβ-injected rat hippocampus, the reduction of circulating fibrinogen with ancrod administration was found to stabilize BBB function as demonstrated by significant decreases in parenchymal fibrinogen [15]. The ancrod treatment was also shown to block IgG (immunoglobulin) permeability into brain consistent with diminished BBB permeability. Overall, lowering fibrinogen acts to inhibit BBB damage. Consideration on the effects of modulating fibrinogen levels, by pharmacological or genetic means, on microglial response is presented in Section 3.1.

A critical point arising from the animal model work was whether human AD brain would also present evidence for fibrinogen extravasation through an abnormal BBB. This question was addressed [15] who examined fibrinogen staining in the entorhinal cortical region in AD and ND (non-demented) individuals. The results showed marked fibrinogen ir outside of microvessels in AD, but not ND, samples. AD brain tissue also exhibited areas of diffuse fibrinogen staining in association with deposits of Aβ. The peptide showed localization to microvessels and also to regions of brain parenchyma. Evidence for leakiness of BBB in AD brain was also found from IgG permeability through the damaged barrier which was absent in ND samples. Overlaps of IgG ir with deposits of Aβ were also evident. Overall, a similar pattern of fibrinogen extravasation through a weakened BBB was evident in human AD brain tissue consistent with the findings from the peptide-injected animal model. 

Fibrinogen infiltration has been documented in frontal cortex and hippocampal regions of AD and ND brain tissue [18]. Both regions showed marked increases in levels of the plasma protein in AD, compared to ND, brain. Extensive regions of fibrinogen immunoreactivity (ir) were observed external to vessels in perivascular and in parenchymal regions. In the latter case, “clouds” of fibrin were reported [18], which likely correspond to the diffuse staining patterns of the plasma protein evident in AD brain samples [15]. The patterns of staining also included fibrin internalized in microvessels (see below) and possibly acting to block CBF. Areas of BBB damage in AD brain are reported sites for plasma protein entry via microhemorrhage [23]. As noted below, the presence of amyloid peptide can modify, and possibly amplify, the effects of fibrin(ogen) as a pathogenic stimulus.

### 2.2. Fibrinogen and Abnormal Fibrin Clots 

The interaction of fibrin(ogen) with Aβ acts to enhance the aggregation of fibrin and the formation of abnormal fibrin clots [20,21]. In essence, the presence of Aβ can result in fibrin clots which are not fully degraded by enzymatic activity from plasmin. The result is a resistance and longevity of residual fibrin clots. An additional possibility is that levels of thrombin may be elevated in AD brain which would act to further increase clot formation (discussed below). In essence, fibrin(ogen) leaking through a damaged BBB is a target for modulation by Aβ. It seems likely that the presence of fibrin clots could alter the deposition of Aβ and its overall clearance from brain.

The aggregation of Aβ in blood vessel walls could modify cerebral amyloid angiopathy (CAA). The effect of fibrin clots on this process has been examined using several transgenic animal models [14,21]. The findings showed that diminishing fibrinogen levels in AD animals had a significant effect to lower CAA. The results suggested that CAA had some dependence on fibrin(ogen) deposits and BBB damage. Importantly, abnormalities in fibrin(ogen) processes contributed to amyloid pathology and neuroinflammation in AD. An outcome from the studies was that blocking the interaction of fibrinogen with amyloid peptide could serve as a means to reduce BBB leakage and lessen neurovascular damage in AD brain.

### 2.3. Pharmacological Inhibition of Fibrinogen Binding to Peptide

An impetus and background for pharmacological targeting of the binding of fibrinogen with Aβ as a strategy to limit BBB and cerebral dysfunction in AD brain has been considered above. A compound selective for this interaction was subsequently identified (RU-505) through high-throughput screening [24]. When applied to transgenic mice, RU-505 was effective in reducing clot formation. Importantly the compound was without effect in altering normal cerebral function in control animals. Transgenic, but not control, animals exhibited regions of cerebral microvessels containing Aβ deposits, suggesting the presence of CAA. These deposits of peptide were markedly reduced with treatment of mice with RU-505. Overall, RU-505 application had a significant effect to reduce BBB damage and cerebral dysfunction by acting to inhibit the interaction between fibrinogen and amyloid peptide. As discussed below in the consideration of the neurovascular unit, the compound RU-505 also has demonstrated efficacy in reducing neuroinflammation and increasing cognitive performance in AD animals.

### 2.4. Fibrin(ogen) and Coagulation Pathways

The positive effects of reducing fibrin(ogen)-dependent neurovascular damage in AD prompted subsequent work to examine coagulation processes in diseased brain. Specifically, independent of direct peptide binding to fibrin(ogen), Aβ deposits were also found to interact with circulating plasma protein factor XII [18,22,25]. The interaction of Aβ with the contact activation system was found to modulate thrombosis resulting in an increase in the spatial extents and duration of fibrin clots. As noted above, an enhancement in clot formation could act to negatively impact the process of CAA and diminish CBF.

The activation by β amyloid of contact system also has an effect on an inflammatory component of the system. In this case, the presence of Aβ can increase the release of the inflammatory mediator, bradykinin [22]. The depletion of the coagulation factor XII was found to have a two-fold efficacy in blocking neurovascular damage. First, the procedure significantly inhibited the deposition of fibrin in brains of transgenic mice [26]. Secondly, the results also suggested that the reduction of factor XII could lessen abnormalities in BBB due to the inhibition of bradykinin actions on cerebral vasculature. It can be noted that anticoagulant treatment has been previously reported to have efficacy in ameliorating effects of Aβ deposition in transgenic AD mice [27,28]. The overall effects of the depletion of factor XII on elements of the NVU are discussed in more detail below.

## 3. Fibrinogen/Fibrin Interactions within the Neurovascular Unit

### 3.1. Fibrinogen/Fibrin Interactions with Microglia

It can be noted that considerable work has examined fibrinogen actions in multiple sclerosis (MS) and animal models of the disease [29,30]. BBB dysfunction, fibrinogen infiltration and microglial activation are characteristic features of MS [31,32] which demonstrates entry of T cells and marked demyelination [33]. Results from MS studies are not considered in this review. The studies cited in this sub-section are relevant to interactions of fibrinogen with microglia in AD brain and animal models of the disease.

Considerable vascular remodeling and increased permeability of BBB are evident following injection of Aβ42 into rat hippocampus [15,16]. As noted above, this animal model also demonstrates regions of fibrinogen in association with activated microglia in parenchyma [15]. The levels of fibrinogen and microgliosis were found to progressively increase from 1 to 7 d post-peptide injection. Regions exhibiting fibrinogen ir were commonly associated with clusters of Aβ, thus patterns of staining in rat hippocampus showed overlap between fibrinogen, peptide deposits and the presence of microglia. The immune cells exhibited an activated ameboid-type phenotype. Controls (injection of PBS or reverse peptide) exhibited little or no evidence for fibrinogen infiltration into parenchyma. Microglia in control presented with a predominantly ramified shape with extended cellular processes typical of a surveillance mode.

The treatment of Aβ42-injected rats with the defibrinogenating compound, ancrod, inhibited microgliosis in addition to markedly reducing levels of the plasma protein [15]. This result suggested a link between immune cell response and fibrinogen permeability through a weakened abnormal BBB. To check this point, anti-Mac-1 (antibody for CD11b) was used to reduce microglial activation. Administration of anti-Mac-1 to peptide-injected animals decreased microgliosis and infiltration of fibrinogen through the BBB. The permeability of IgG into brain parenchyma was markedly diminished indicating the antibody treatment was effective in reducing BBB damage. The use of anti-Mac-1 to modulate ROS in AD animals is discussed below (Section 3.6). Transgenic animal studies [13] have also reported a correlation between fibrinogen infiltration and microglial activation, with ancrod depletion of fibrinogen inhibiting microglial activation and the proliferation of cells.

The entorhinal cortex in AD brain tissue was also examined for association of fibrinogen and immune cell response [15]. AD human brain exhibited considerable areas of overlap between fibrinogen and microglia, a pattern of staining absent in ND tissue. Microglia were characterized by an ameboid morphology, suggesting an activated state similar to that found in Aβ42-injected rat brain. Subsequent work using AD and ND brain samples has shown a close association between clusters of microglia and fibrinogen in parenchymal regions of the diseased brains [34]. Overall, human AD brain demonstrates a continuum of pathology including abnormalities in blood vessels, a decreased intactness of BBB, deposits of Aβ and the presence of clusters of activated and proliferating microglia often co-localized with peptide deposits.

Neuroinflammatory responses mediated by microglia showed a time-dependent correlation with activation of the coagulation contact system in cortical and hippocampal areas of transgenic mice [26]. The depletion of plasma factor XII significantly reduced microgliosis in the AD animals. Concomitant measurements showed that fibrin deposition was also decreased in animals with depleted factor XII. The authors suggested that a damaged BBB could allow leakage of contact system components and bradykinin into AD brain. The neuroinflammatory factor bradykinin is produced from contact system activation and can act on cells of NVU including microglia. Overall, contact system response, modulated by the presence of Aβ, is linked to microglial activation and neuroinflammation in transgenic animals.

At present, the involvement of Aβ as a direct effector of microglial response [35] or as mediated by peptide interactions to modify fibrin(ogen) and contact system factors is complex and not well understood in relation to AD pathology. It can be noted that a detailed review of the mechanisms by which microglia express and produce ROS in AD is available [36]. However, the review does not include aspects of fibrin(ogen) as an overall pathogenic stimulus in AD brain.

### 3.2. Fibrinogen/Fibrin Interactions with Astrocytes

The proximity of astrocytic end-feet to BBB would suggest involvement of the cells in response to fibrinogen extravasation in AD brain. Furthermore, and relevant to this review, activated astrocytes can secrete a spectrum of neurotoxic species including ROS with stimulation by amyloid-β peptide [37,38]. At present, data are limited concerning astrocytic involvement in fibrinogen entry in AD brain. However, intra-hippocampal injection of Aβ42 in rat brain significantly increased the area density of GFAP (+) astrocytes relative to animals receiving PBS injection [15]. Some localization of astrocytic staining in proximity to infiltrating fibrinogen was observed. Ancrod administration to reduce levels of fibrinogen blocked astrogliosis in the peptide-injected animals. The reduction of plasma factor XII in the contact system has also been reported to lower astrogliosis in transgenic AD animals [26]. These results prompted authors to comment that contact system response is coupled to astrocyte activation (and microglial activation as noted above) which could increase neuroinflammation in AD brain.

AD human brain tissue demonstrates increased astrogliosis relative to astrocytic response in ND samples [15]. This work also reported evidence for patterns of double staining (fibrinogen/GFAP) showing co-localization of fibrinogen with activated astrocytes in parenchyma. In vitro work using co-cultures of astrocytes and neurons has established that the Aβ-stimulated glial cells are toxic to neurons. The results suggested astrocytic oxidative stress induced by activation of NADPH oxidase [37]. Future work will be required in the clarification of astrocytic involvement and response to abnormalities in BBB and specifically how cellular interactions with fibrinogen contribute to inflammation in the progression of AD.

### 3.3. Fibrinogen/Fibrin Interactions with Neurons/Synaptic Connections

The effects of a fibrinogen-permeable BBB on neuronal viability have been examined in the Aβ42 intra-hippocampal injection animal model [15]. The numbers of neurons in the granule cell layer were reduced relative to rats receiving control (PBS) injection. The treatment of peptide-injected animals with ancrod to lower fibrinogen levels was found to confer significant neuroprotection. This result suggested that fibrinogen extravasation into brain contributed to neuronal loss. A fibrinogen-induced component of neurotoxicity was also supported by the finding that the injection of fibrinogen with Aβ42 further increased loss of granule cell neurons compared with peptide injection alone. Interestingly, Aβ42-injected animals receiving administration of anti-Mac-1 antibody to reduce microglial activation showed an enhancement in numbers of neurons. This neuroprotective strategy has since been used in subsequent work and is discussed below in Section 3.6.

The presence of fibrinogen in areas populated with dystrophic neurites has been reported in AD brain [18]. This pattern of fibrinogen parenchymal staining was absent in samples from ND individuals. This group also used a transgenic animal model to examine for effects of fibrinogen infiltration on neuronal viability in the subiculum area of mouse hippocampus. The results showed that transgenic animals, treated with a monoclonal antibody to reduce fibrinogen levels, had a significant increase in numbers of viable neurons relative to untreated mice. Overall, AD animals demonstrated considerable areas of fibrinogen staining external to blood vessels with evidence for localization to deposits of Aβ and to damaged neurites.

The depletion of coagulation factor XII has been found effective to protect against neuronal damage in AD animals [26]. In addition, cognitive function was improved with the depletion of the factor which drives the contact system response. Transgenic mice treated to lower factor XII exhibited reduced levels of fibrin deposition relative to untreated animals. The results were consistent with an increased neuronal viability due to a reduction in fibrin clots. However, as noted above, the depletion of factor XII also inhibits bradykinin release from the inflammatory component of the contact system. Thus, contact system activation-mediated increases in fibrin clots and bradykinin could contribute to neurodegeneration in the transgenic animals.

### 3.4. Fibrinogen and Endothelium

Endothelial cells (EC) are perturbed in AD brain [5,39]. However, the changes in molecular properties underlying endothelial cell dysfunction associated with fibrinogen infiltration from plasma have yet to be determined in AD animal models. In addition, the specific abnormalities in EC associated with overall leakiness of BBB are not well understood. Breakdown of BBB and loss of cortical endothelial tight junctions have been documented in AD brain [40,41]. The extravasation of fibrin(ogen) provides a pro-inflammatory stimulus to alter EC properties. As one example, fibrin(ogen) acts as a potent stimulator of the inflammatory chemokine IL-8 in EC [42]. The CXCR2 receptor for IL-8 is highly upregulated in human AD, compared with ND, brain tissue and in an animal model of AD [43]. This chemokine also exhibits the largest increase in expression of any factor in microglia stimulated with Aβ42 [44]. Overall, fibrin(ogen) entry into brain could induce a spectrum of inflammatory factors in the NVU causing abnormalities in EC function [30].

Although data correlating abnormalities in EC with fibrin(ogen) entry into brain are limited, the effects of thrombin on EC have been examined [45]. Indeed, EC synthesize thrombin in AD brain [46] and, as noted above, thrombin mediates the formation of fibrin via the coagulation process. A number of studies have linked thrombin and endothelial cell activation with AD neurovascular damage [39]. Endothelial cells derived from AD, but not ND, individuals synthesize considerable amounts of the protease which can act to damage cells of the NVU [46]. Furthermore, endothelial cells subjected to oxidative damage have been shown to secrete high levels of thrombin [47]. Thrombin activation of endothelium increases a milieu of pro-inflammatory factors, promotes leakiness of BBB and induces chronic inflammation in AD. Overall, thrombin as a paracrine or autocrine factor for EC, or via its protease action to increase levels of fibrin, could contribute to a neurotoxic environment in AD brain.

### 3.5. Fibrinogen and Pericytes

Functional roles for pericytes in health and disease have been reviewed [48]. At present, there are sparse data linking fibrinogen entry into parenchyma with loss of pericyte function in AD animal models [49]. Overall, the changes and perturbations in pericyte function in AD comprise a relatively understudied area of research [50,51]. A reduction in overall number of cells and deficiency in function of pericytes has been reported in AD [52]. The loss of pericytes was correlated with abnormalities in BBB function and fibrin deposition in cortical and hippocampal regions of AD brain [53]. This work also reported patterns of extravascular IgG further suggestive of a weakened BBB. In a study with no specific links to AD, mice incorporating multiple gene mutations were used to reduce the number of functional pericytes [54]. The mutated animals exhibited a breakdown of BBB, allowing infiltration of fibrinogen into cortical and hippocampal regions of brain. 

Although pericyte loss is associated with a leaky BBB to albumin and IgG, fibrinogen is a unique pathogenic stimulus in AD brain as a precursor to fibrin aggregation and clots and through induction of inflammatory reactivity. At present, roles of Aβ in the modulation of pericyte response to fibrinogen activity are not well understood. A basic question concerns relationships between BBB damage, fibrinogen leak and effects on pericyte communication with other cells of the NVU. Experiments using pharmacological or genetic tools to manipulate levels of fibrinogen will have utility to examine correlation and coupling of the plasma protein entry into brain with pericyte dysfunction and loss.

### 3.6. Fibrinogen and Cell-Derived Reactive Oxygen Species

The studies cited above link fibrinogen invasion through a weakened BBB to responses from cells in the NVU ensemble. However, the mechanisms of actions which underly cellular interactions with fibrin(ogen) have generally not been addressed in these studies. A rationale from previous work [15] was that CD11b in microglia could serve as a pharmacological target for modulating fibrinogen effects in AD brain. The work discussed below follow this strategy. Importantly, fibrinogen binding to the CD11b receptor has been found to activate NADPH oxidase and subsequent cellular release of ROS [55].

Recent work has led to the findings that oxidative stress may play an important role linking the entry of fibrinogen through a defective BBB and subsequent neuronal and synaptic damage in AD brain. One study has used the animal model 5XFAD which expresses mutations of amyloid precursor protein and presenilin [56] with relevance to familial AD. Mutant mice demonstrate a weakened BBB and fibrin clusters localized to peptide deposits. The objective of the work was to block oxidative stress resulting from fibrin stimulation of microglia. The study used a fibrin-binding monoclonal antibody to block fibrin interaction with CD11b microglial receptor. The findings showed that microglial activation was inhibited with antibody treatment and that NADPH oxidase activation and subsequent cellular ROS production was markedly reduced. The authors concluded that their strategy to block oxidative stress, mediated by fibrin(ogen)-stimulated microglia, provided neuroprotection in the AD animal model.

Another study by the same group has examined fibrinogen involvement in dendritic spine elimination in AD animals and in mice receiving fibrinogen injection [57]. The work first documented fibrinogen expression in 5XFAD cortical and hippocampal regions with dendritic damage associated with fibrinogen deposits. AD human brain samples generally replicated the animal findings. Neurovascular dysfunction was also examined in the cortex of mice receiving fibrinogen injection [57]. The results showed a change in microglial phenotype to an activated state in response to the plasma protein injection. Loss of dendrites and spine was associated with the microglial response and activation of cellular release of ROS. The NADPH inhibitor apocynin was protective against fibrinogen-induced damage.

The effects of genetic disruption of CD11b binding on cognitive performance were also investigated [56]. Mice with no intact CD11b receptor site for fibrinogen showed improved learning and memory tasks compared with AD animals. Animals lacking CD11b-mediated fibrinogen activation demonstrated reduced microglial activation, neuronal damage and peptide deposition relative to control mice. The authors concluded that microglial activation, induced by fibrinogen binding to CD11b, leads to cellular secretion of neurotoxic ROS. This finding could account for the loss of neurons in peptide-injected rat hippocampus [15] and in transgenic animals [18].

## 4. Conclusions

Considerable experimental evidence has linked abnormalities in BBB with the early and progressive loss of memory and cognitive function which characterize AD brain. One such abnormality is the leakage of fibrinogen into brain parenchyma and the subsequent effects of the plasma protein to activate cells comprising the NVU. In essence, a perturbed and compromised BBB contributes to the underlying neurodegeneration present in diseased brain [58,59]. Overall, the mechanisms linking BBB leakiness to fibrinogen with neuronal and synaptic dysfunction are not well understood [1,32,60]. Perturbations in vascular function likely constitute a component of multifactorial abnormalities in AD pathology.

The passage of plasma protein fibrinogen into brain is associated with a multiplicity and complexity of effects on cells of the NVU. Furthermore, the presence of Aβ acts to modify the effects of a leaky BBB in AD brain. The transition of fibrinogen to fibrin clots is altered by peptide such that fibrin aggregation and clots are modified to abnormal forms which resist enzymatic degradation. Fibrin(ogen) in brain parenchyma appears in association with deposits of peptide. As noted below, immune cell response to the association of these two stimuli could amplify and sustain neuroinflammation in AD.

The presence of Aβ also acts to modify the coagulation process through interaction with circulating plasma factor XII of the contact system. The result is increased thrombin and the subsequent formation of fibrin aggregation within microvessels and perivascular regions. Activation of the inflammatory arm of the contact system leads to elevated levels of bradykinin. In addition, the presence of soluble Aβ deposits in vessel walls and perivascular areas could alter the process of CAA and cause disruption of BBB. It is likely that the formation of abnormal fibrin clots may also act to occlude microvessels and directly compromise CBF. NVU endothelial cells and pericytes are putative cellular targets for dysfunction with exposure to the different processes.

**Figure 1:**
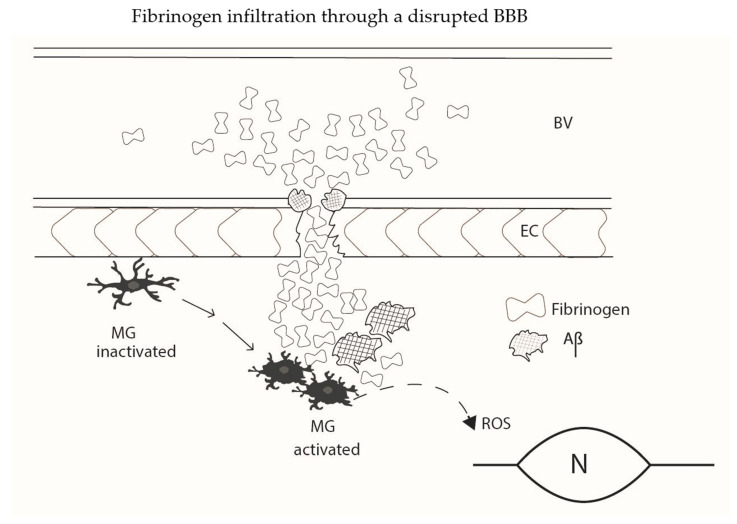
A simplified schematic diagram illustrating aspects of fibrinogen extravasation through an abnormal BBB in AD brain. Clusters of plasma fibrinogen are associated with a damaged endothelium and permeate into brain parenchyma. The aggregation of fibrin(ogen) is modified by the presence of Aβ resulting in formation of abnormal deposits of fibrinogen, fibrin and Aβ. Inactivated microglia (MG) in surveillance mode are mobilized to an activated phenotype (MG activated) in response to fibrin(ogen)-Aβ stimulation. Microglial activation is coupled to cellular ROS production which is toxic to bystander neurons and synaptic connectivity. Additionally shown are soluble Aβ deposits in microvessel walls in association with fibrin(ogen) with possible involvement in the process of cerebral amyloid angiopathy (CAA). The effects of CAA and the actions of plasma fibrin accumulation to block cerebral blood flow (CBF) could also contribute to neuronal and synaptic damage. Interactions of Aβ with the contact system in the coagulation process and interactions involving NVU members, astrocytes and pericytes are not shown. BV (blood vessel); EC (endothelial cell; MG (microglia); N (neuron).

A schematic diagram summarizing elements of fibrinogen infiltration through a leaky BBB is presented (Figure 1). The diagram shows a diversity of effects of Aβ to modulate responses in AD as noted above. Microglia are key cells in initiating or responding to vascular perturbations such as an increased permeability of BBB to plasma protein. Fibrinogen extravasation through a damaged BBB, and modulation of fibrin(ogen) by Aβ deposits, serves as an unique microglial activation stimulus in AD brain. The microglial release of ROS appears as a critical endpoint process linking fibrinogen infiltration and its complexity of effects with neuronal and synaptic damage. The simplified diagram does not include cells of the NVU, such as astrocytes or pericytes, or aspects of the plasma coagulation process such as contact system activation.

ROS released from other cellular sources could also contribute to neurotoxicity in conditions of a disrupted BBB. As noted above (Section 3.2), β-amyloid peptide has been reported to induce oxidative stress in astrocytes with the activated glial cells releasing ROS leading to neuronal damage [37]. Activated pericytes are also reported to cause ROS-mediated neurodegeneration [61]. Microglial-derived ROS, in response to fibrin(ogen) stimulation, could damage other NVU members including pericytes and endothelium [62]. Animal model results also suggest reactive nitrogen species from Aβ-stimulated microglia may be involved in leakiness of BBB and cellular damage [63]. Overall, activation of microglia with fibrin(ogen)-Aβ stimulation could yield a milieu of pro-inflammatory paracrine factors toxic to bystander cells of the NVU. Autocrine products abundantly released from microglia, such as IL-8 and TNF-α, could act to amplify and sustain cellular responses in association with a damaged BBB in AD brain. 

AD is considered a multifactorial disease and evidence indicates that BBB disruption is a component factor. At this time, correlative data are lacking to state that fibrinogen infiltration through a weakened BBB is a causative factor in disease etiology. Vascular dysfunction is intertwined with AD pathology in a complex manner. As one example, the interaction of fibrin(ogen) with Aβ leads to aggregates of the two which persist in brain parenchyma. These abnormal deposits could exacerbate reactive microglial proinflammatory response linking fibrinogen permeability into parenchymal regions to pathological processes in AD. The diverse aspects of vascular dysfunction, with particular relevance to loss of cognition in AD, have been considered [12]. At present, a number of studies have identified BBB breakdown as an early characteristic property associated with cognitive dysfunction [10,11,58]. However, few studies have examined fibrinogen extravasation as a mechanism causing a decline in cognitive performance.

Future work is required to better understand the links between a dysfunctional BBB, neuroinflammation and neurodegeneration. Animal model and in vitro studies will have utility in addressing how Aβ-modified fibrinogen and fibrin aggregation and clots act on members of the NVU. As one example, at present, there are little experimental data concerning pericyte responses to the formation of abnormal fibrin clots. Questions pertinent to the effects of peptide-altered fibrin(ogen) aggregation on magnitudes and persistence of chronic inflammation in AD need study. Pharmacological manipulations, such as altering levels of fibrinogen, binding of the plasma protein with Aβ, and interactions of fibrin(ogen) with microglia, will be useful in the interpretation of processes involved in a fibrinogen-permeable dysfunctional BBB. Elaboration of the interactions between NVU cells and effectors causing dysregulation of BBB will allow testing of neuroprotective strategies for neurons and synaptic connectivity in diseased AD brain.
